# Enhancing letter recognition in first graders with embedded picture mnemonics: insights from cognitive load and dual coding theories

**DOI:** 10.3389/fpsyg.2026.1726843

**Published:** 2026-03-19

**Authors:** Barış Kalender

**Affiliations:** Department of Primary Education, Faculty of Education, Gaziantep University, Gaziantep, Türkiye

**Keywords:** extraneous cognitive load, cognitive load theory, dual coding theory, embedded picture mnemonic, letter recognition, Turkish alphabet

## Abstract

**Introduction:**

This study aimed to evaluate the effectiveness of embedded picture mnemonics, specifically designed for the Turkish alphabet, in improving letter recognition skills among first-grade students. The research was grounded in two prominent cognitive frameworks: Cognitive Load Theory and Dual Coding Theory. While Cognitive Load Theory suggests that reducing unnecessary cognitive effort enhances learning, Coding Theory posits that combining verbal and visual information strengthens memory and comprehension.

**Methods:**

A quasi-experimental design was used, with participants divided into three groups: an experimental group (*N*=30) and two control groups (*N*=25 and *N*=30). Letter recognition accuracy and speed were assessed using a multiple-choice test administered via tablets, and data were analyzed using 2x3 doubly multivariate repeated measures analysis of variance (RM-MANOVA).

**Results:**

Multivariate analysis revealed a significant main effect for the intervention group, with the experimental group significantly outperforming both control groups. Specifically, the mnemonic-based instruction yielded large effect sizes for both letter recognition accuracy (*F*(2, 82) = 11.68, *p* < 0.001, η*p*^2^ = 0.222) and recognition speed (*F*(2, 82) = 22.59, *p* < 0.001, η*p*^2^ = 0.355). Descriptive statistics also showed that students in the experimental group achieved higher letter recognition accuracy and faster recognition speed in both measurement points.

**Conclusion:**

The results are consistent with theoretical assumptions derived from Cognitive Load Theory, suggesting that embedded picture mnemonics may support more efficient processing of letter–sound correspondences. Moreover, the use of visual associations supported by dual coding enhanced students’ ability to recognize and recall letters. These findings align with prior research and highlight the potential of mnemonic-based strategies in early literacy instruction within similar linguistic and instructional contexts.

## 1 Introduction

Multiple approaches exist for introducing early reading skills to first-grade learners, and these have been categorized in diverse ways according to various pedagogical perspectives (Çelenk, 2002; Aukerman, 1971). When examining methods of teaching reading based on their part-whole relationships, they can be categorized into three approaches according to Akyol (2005): 1. Part-to-whole approach, 2. Whole-to-part approach, and 3. Interactive approach. In Turkey, the “Phonics-Based Early Literacy Instruction” situated within the part-to-whole approach to reading instruction for first-graders, has been implemented since 2004 [MoNE (Milli Eğitim Bakanlığı), 2018]. This method entails conducting the reading and writing process as outlined by the Ministry of National Education (MoNE) in 2018:

1- Introduction phase for reading and writing

a Preliminary listening drills
b Stimulating fine motor skills
c Painting and drawing exercises

2- Progressive phase

a *Perceiving, recognizing, and distinguishing the phonics*
b Reading and writing the letters
c Forming syllables, words, and sentences
d Reading texts

3- Self-guided reading phase

According to the aforementioned stages, the Phonics-Based Early Literacy Instruction Method begins with the preparation of the necessary psychomotor and cognitive skills for reading and writing. In the subsequent stages, in accordance with the stages mentioned by Armbruster et al. (2001), activities are carried out to audio perception, recognition, and distinguishing the phonics of the alphabet, followed by activities for recognizing the symbol (letter) of each phonic. The aim is to enable the student to perceive, recognize, and distinguish the phonics. At this point, it is necessary to explain the skills of phonological and phonemic awareness, which involve perceiving, recognizing, and distinguishing the phonics. Perceiving, recognizing, and distinguishing the sound are expressed as the use of phonological and phonemic awareness. Such an awareness is crucial in the reading and writing processes since they serve as a prerequisite condition for recognizing and distinguishing letters.

More specifically, phonological awareness involves noticing the sound units that comprise of a word (Goswami and Bryant, 2016), recognizing those sound units (Gray and McCutchen, 2006), distinguishing and manipulating them (Anthony and Francis, 2005), and consequently, being able to break down words and syllables expressed in language into smaller subunits (Allor, 2002; Denton et al., 2000). On the other hand, phonemic awareness is the ability to focus on each individual sound within phonological awareness and to perceive them (Chard and Dickson, 1999). Recent empirical studies in early literacy and foreign alphabet learning have demonstrated that embedded picture and multimodal mnemonics significantly enhance letter–sound mastery, reduce confusion, and improve short-term retention (Shmidman and Ehri, 2010; Matsunaga, 2003). These researchs’ results suggest that mnemonics can concretely support phonological and phonemic awareness development in young learners. In this context, it can be stated that phonological and phonemic awareness are critically important at the stage of perceiving, recognizing, and distinguishing sounds, which is essential in the whole reading process. This study aims to demonstrate the effect of using mnemonics to facilitate the perception, recognition, and differentiating sounds.

### 1.1 Literature review and theoretical framework

In this study, the Dual Coding Theory and Cognitive Load Theory have been employed as the theoretical framework for investigating mnemonics applied to the triple stage of perceiving, recognizing, and distinguishing the particular sound.

#### 1.1.1 Dual coding theory

During the process of perceiving and recognizing sounds, teachers typically provide both a verbal stimulus (the name of the object representing the sound) and a non-verbal stimulus (the visual representation of the object) simultaneously, enabling the processing of the two different stimuli to provide a connection between them (Akyol and Temur, 2008). The associative connection to be established between these two units facilitates retrieval (Clark and Paivio, 1991, p. 152). Dual Coding Theory posits that human memory is organized into two interconnected systems: a verbal system for linguistic information and a non-verbal system for perceptual imagery (Paivio, 1986). Thus, the reading process involves introducing the sound of the letter introduced into the verbal system, accompanied by showing an object beginning with the sound to fully represent the letter. Moreover, combining visual and auditory elements in mnemonic strategies, such as picture plus sound mnemonics (Matsunaga, 2003) and interactive illustrations (Brigham and Brigham, 1998), has been shown to improve immediate recall and facilitate associations between verbal and non-verbal systems. This approach aligns with the dual coding principle of establishing within- and between-system connections to strengthen memory retrieval (Clark and Paivio, 1991).

Within this theoretical framework, when introducing the sound “e,” the name of an object that begins with this letter, such as “elma” (meaning ‘apple’), is articulated, and its illustration 🍎 is demonstrated simultaneously. Subsequently, the letter representing the sound is symbolically presented, along with both the spelling and the illustration of objects beginning with this letter (Kalender, & Guleç, 2021). This instructional practice can be theoretically explained within the framework of Dual Coding Theory, as it promotes the formation of associative links between verbal and non-verbal representational systems.

**Figure 1:**
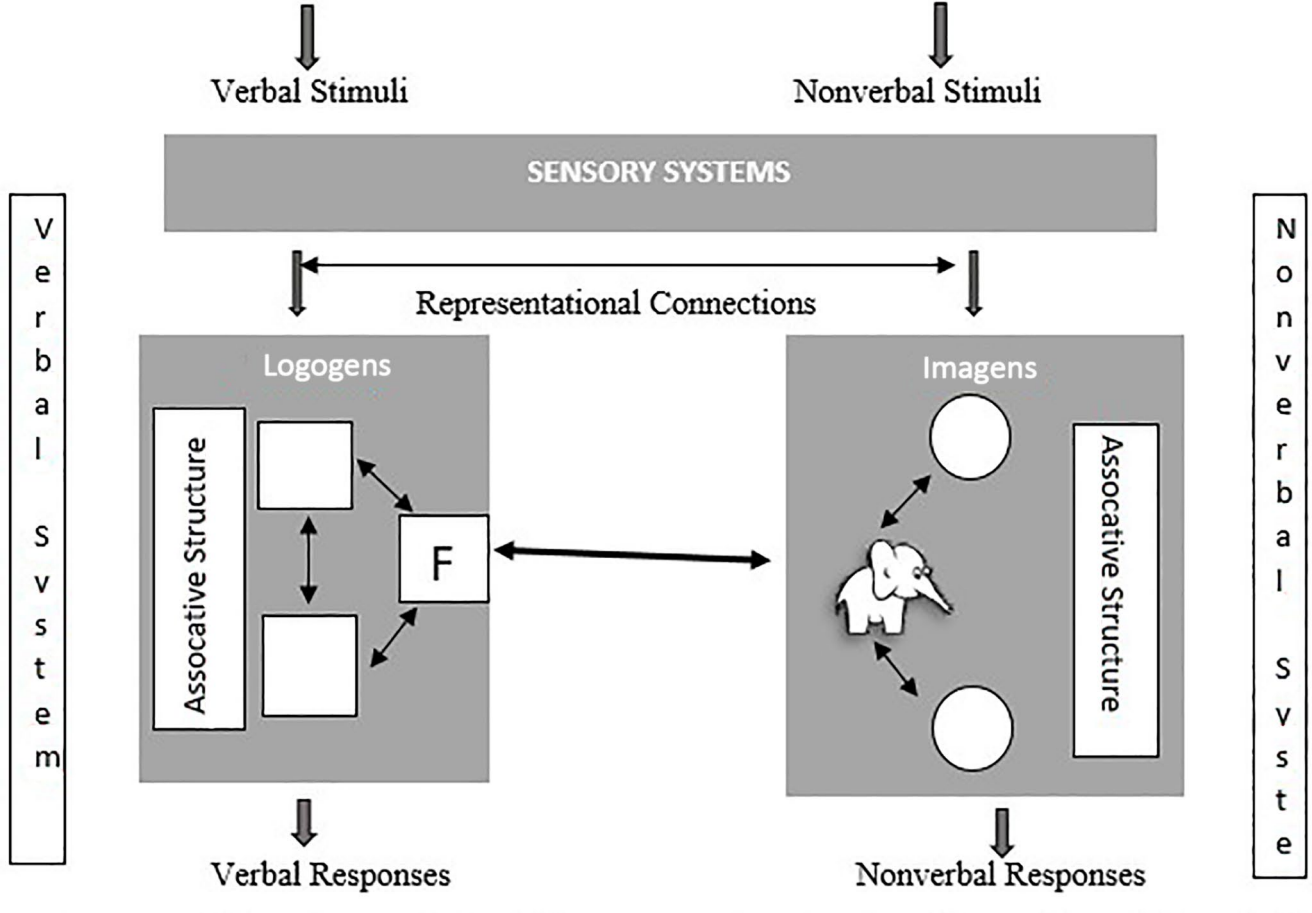
Verbal and nonverbal symbolic systems of Dual Coding Theory [adapted from Clark and Paivio, 1991, p. 151]. The bold arrow represents between-systems (referential) connections, and thin arrows represent with-in system (associative) connections in this figure and Figure 2.

According to Paivio’s (1986) Dual Coding Theory, learning involves interactions between verbal and non-verbal representational systems. If stimuli for each subsystem increase, associative connections are further enhanced by establishing within-system and between-system connections. Based on the theory, facilitating associations by establishing within-system and between-system connections make recalling easier for the learner (Paivio, 1991). The implementation of the reading process for first-grades in the current practice in Türkiye is described in Figure 1, as mentioned earlier (e.g., the example of an apple).

As shown in Figure 1, the student is provided both with the auditory stimulus for the letter “F” and a visual of an accompanying elephant (fil) to introduce the letter “F.” The “F” sound is processed in the verbal system, while the image serves to establish an within-system associative connection in the non-verbal system. After the within-system connections (referential) occur independently in both systems, in essence efforts are directed to establish between-system connections through these two stimuli. With the establishment of these connections, the stages of perception, recognition, and distinguishing the sound in Phonics-Based Early Literacy Instruction Method is completed. Upon completing this stage, the letter “F” is introduced as a sound and presented visually. The student is then encouraged to establish a connection between the visual representation of the letter and the auditory stimuli of the letter “F” encountered earlier, along with the visual stimulus of an elephant encountered in the non-verbal system. This is believed to facilitate the transition of the student to the stage of reading the letter.

**Figure 2:**
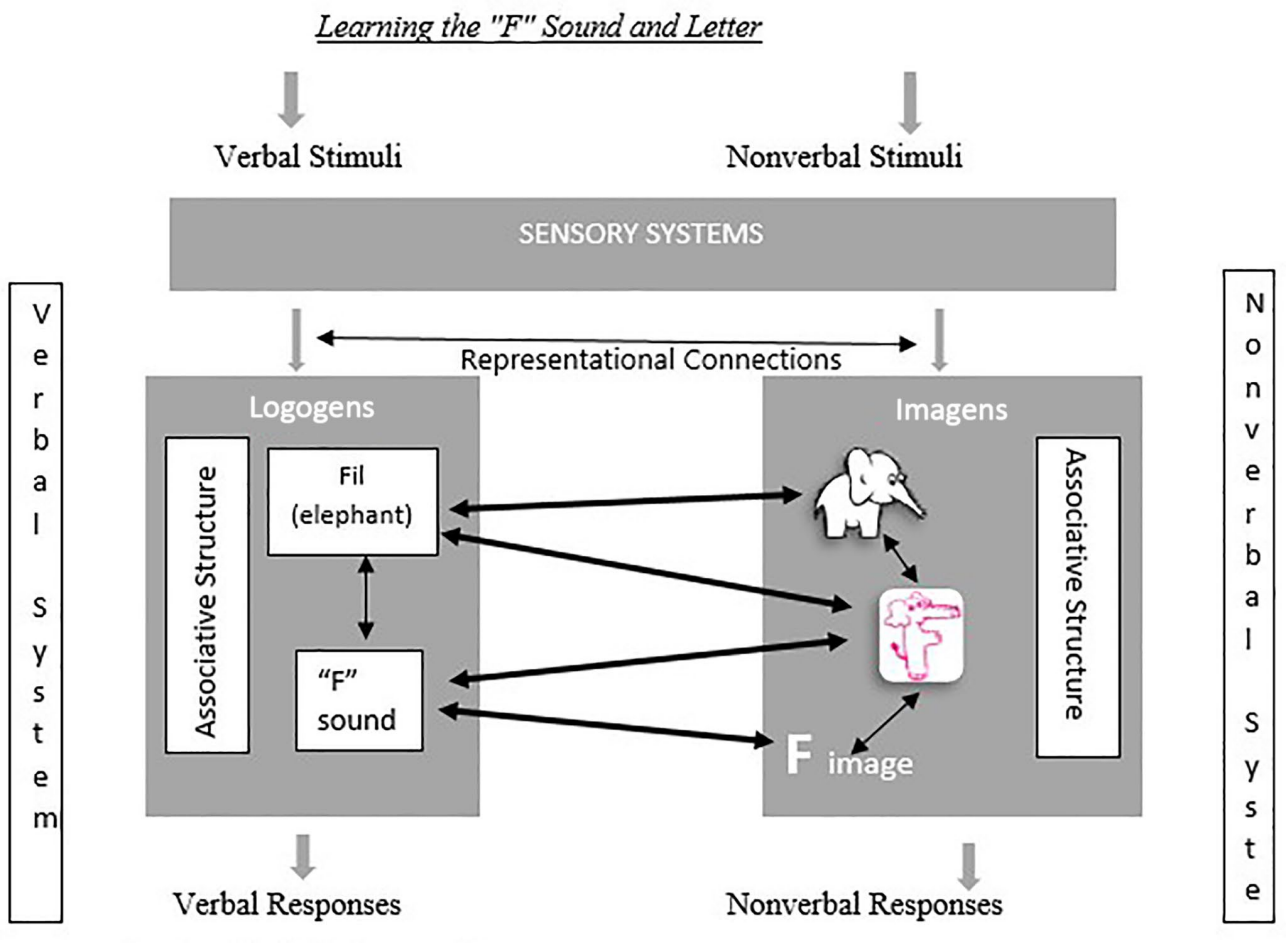
Function of memory with mnemonic illustrations. Source: (Kalender, & Guleç, 2021).

Although this conventional approach establishes basic referential connections, it still requires learners to coordinate multiple representations sequentially. To address this limitation, the present study adopts an embedded mnemonic design, as illustrated in Figure 2.

In Figure 2 it is evident that the mnemonic prepared for the “F” sound establishes a within-system connection with the “elephant/fil” visual in the non-verbal system and a between-system connection with the word “elephant” in the verbal system. With this mnemonic, when the word “elephant” is pronounced, the “F” sound is emphasized, enabling the student to perceive and distinguish this sound. Following this stage, when the letter “F” is visually presented, the student is encouraged in recognizing and recalling the sound by establishing an association it with the mnemonic introduced in the previous stage due to its resemblance. In this particular context, it can be asserted that the mnemonic devised for the “F” phoneme affords an avenue for forging a within-system association, whereby one constituent, the familiar visual depiction of an elephant, intersects with the novel visual representation of the letter “F” within the verbal framework. Moreover, this mnemonic device facilitates the establishment of a between-system correlation, giving way to the connection between the verbal and non-verbal domains, as well as fostering the integration of word and sound associations. Therefore, it can be concluded that this mnemonic is expected to aid students perceiving, recognizing, and distinguishing the sound with ease.

In line with this reasoning, mnemonic strategies have been shown to support letter–sound correspondence and early decoding skills. McNamara (2012) found that using these tricks helped students recognize letters and know their sounds better, which supports the idea that they help with reading and spelling.

From a cognitive load perspective, as will be elaborated in the following section, referential connections established through embedded picture mnemonics reduce the need for learners to actively search for and mentally integrate verbal and visual information. When the letter form and its associated image are combined into a single representational unit, learners are no longer required to coordinate multiple sources of information, thereby minimizing unnecessary processing demands. In this sense, the referential connections described by Dual Coding Theory directly contribute to a reduction in extraneous cognitive load. Accordingly, this study conceptualizes embedded picture mnemonics as a mechanism through which dual-coded referential connections function as an instructional design tool to minimize extraneous cognitive load during early letter–sound learning.

#### 1.1.2 Cognitive load theory

While Dual Coding Theory explains how mnemonic associations are formed, Cognitive Load Theory provides insight into why such designs may reduce processing demands during early reading instruction. The Cognitive Load Theory, as defined by van Merrienboer and Sweller (2005), suggests the load on memory in the learning process, emphasizing the interaction between knowledge structures and human cognition, irrespective of context. Cognitive Load Theory provides a comprehensive framework for understanding how human cognitive architecture impacts learning processes in educational environments (Paas et al., 2010). According to Cognitive Load Theory, working memory serves as a critical bottleneck during learning, and cognitive load is commonly classified into intrinsic, extraneous, and germane components (Paas et al., 2003a). Intrinsic cognitive load, determined by the difficulty of the subject matter, is inherent and unchangeable (Seufert et al., 2007).

Conversely, extraneous cognitive load arises from poorly designed learning materials and can be mitigated through instructional design (Gerjets et al., 2004). Sweller (2011) also suggests that while intrinsic cognitive load is inevitable, extraneous cognitive load can be reduced through changes in teaching methods. Cognitive load measurement techniques provide essential insights for cognitive load theory by revealing information that traditional performance-based measures might not capture, and the combination of performance outcomes with cognitive load measures is considered a reliable indicator of the mental efficiency of instructional methods (Paas et al., 2003b).

In this study, mnemonics developed within the framework of Dual Coding Theory provide students with an opportunity to link new information—the sound and visual representation of letters—with existing cognitive schemas during the stages of perceiving, recognizing, and distinguishing sounds and letters. Evidence from diverse learner populations—including preschoolers, students with learning disabilities, and university students—indicates that mnemonic illustrations can reduce extraneous cognitive load while enhancing learning outcomes across a range of domains, from early literacy to medical education (Scruggs and Mastropieri, 1991; Hawkins, 2016). These findings suggest a broad applicability of mnemonics in supporting efficient memory processing. By presenting a single, integrated stimulus for learning each letter, mnemonics align with Sweller (2011) recommendation to minimize extraneous cognitive load.

**Figure 3:**
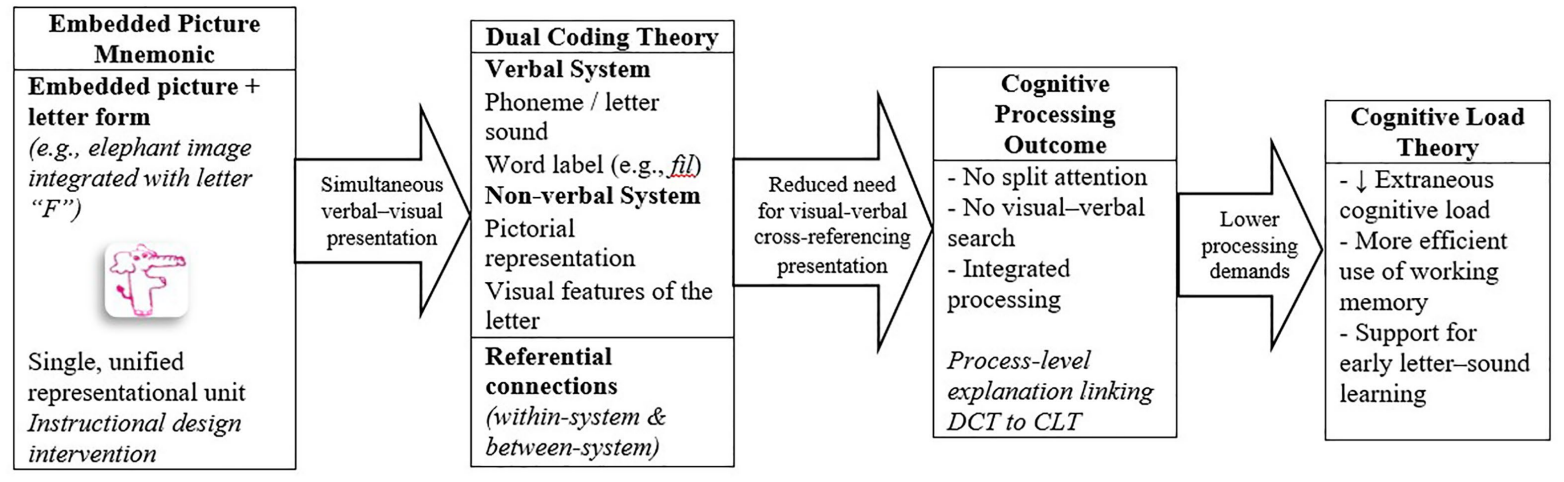
Conceptual model of how embedded picture mnemonics reduce extraneous cognitive load.

Furthermore, the use of mnemonics complements Sweller (2011) “Modality Effect,” which emphasizes the advantage of presenting information through multiple modalities. Recognizing letters is a crucial prerequisite for developing reading skills (de Graaff et al., 2007), and the design of processes containing facilitating materials for student learning is vital. Mnemonics not only stimulate different stimulus systems as per the dual coding theory but also reduce extraneous cognitive load, as suggested by the cognitive load theory. Thus, while Dual Coding Theory explains how mnemonic associations are formed through referential connections, Cognitive Load Theory clarifies why such associations facilitate learning by reducing extraneous processing demands during early reading instruction. This proposed mechanism is conceptually illustrated in Figure 3.

As illustrated in Figure 3, taken together, Dual Coding Theory and Cognitive Load Theory offer a complementary explanation of how embedded picture mnemonics support early letter–sound learning. While Dual Coding Theory accounts for how verbal and non-verbal representations are connected through referential links, Cognitive Load Theory explains why these connections facilitate learning by reducing extraneous cognitive load. Specifically, by embedding the letter form within a meaningful pictorial representation, integrated mnemonics reduce the need for learners to actively search for and mentally coordinate separate verbal and visual sources of information. This instructional design minimizes split attention and unnecessary processing demands, thereby enabling more efficient use of working memory during the stages of perceiving, recognizing, and distinguishing letter sounds.

Across multiple domains—including science, history, and music—mnemonic illustrations, whether researcher-provided or student-generated, have been shown to consistently enhance immediate learning and short-term retention (Mastropieri et al., 1985; Rummel et al., 2003; Brigham and Brigham, 1998). Given that early literacy acquisition relies heavily on establishing accurate and durable letter–sound associations, such findings are particularly relevant to the initial stages of reading instruction. Research conducted in Türkiye consistently indicates that the early reading process is marked by multifaceted and persistent challenges stemming from student-, teacher-, family-, and program-related factors. Qualitative and mixed-method studies have shown that first-grade students frequently experience difficulties in perceiving and discriminating similar sounds, blending letters into syllables, and establishing stable letter–sound correspondences, particularly during the initial stages of literacy instruction (Babayiğit and Erkuş, 2017). Teachers further report that insufficient school readiness, limited preschool attendance, and underdeveloped phonological and linguistic foundations significantly hinder early reading development (Anras, 2020; Sağırlı and Atik, 2022).

Beyond these learner-related factors, structural and contextual constraints—such as crowded classrooms, limited instructional materials, parental misconceptions about literacy instruction, and age-related readiness issues introduced by systemic reforms—have been widely documented as limiting the effectiveness of early literacy instruction (Aktürk and Taş, 2011; Babayiğit and Erkuş, 2017). More recently, studies conducted during the COVID-19 pandemic have demonstrated that disruptions to face-to-face instruction exacerbated existing inequalities, making it particularly difficult to sustain systematic letter–sound instruction through educational technologies and increasing students’ reliance on parental mediation (Atik and Avcı, 2021; Susar Kırmızı and Büyükdağ, 2022). A recent synthesis of postgraduate theses further confirms that, although these challenges have been extensively documented in the Turkish context, proposed solutions largely remain descriptive and practice-oriented, with limited emphasis on theoretically grounded instructional designs targeting early letter–sound learning (Özcan, 2024). Notably, the absence of empirical studies examining mnemonics specifically designed for and applied to the Turkish alphabet highlights a critical gap in the literature. Taken together, this body of research suggests that while the difficulties of early literacy instruction in Türkiye are well established, there is a notable lack of empirically tested, theory-driven interventions—particularly mnemonic designs grounded in cognitive theory—tailored to the perceptual and associative demands of early letter–sound learning in the Turkish alphabet. Taken together, these findings underscore the potential relevance and effectiveness of mnemonics within the Turkish alphabet context, providing a strong rationale for the current investigation. In this study, the researcher aims to examine the effect of mnemonics, previously introduced in 2021, on students’ ability to perceive and recognize sounds and letters during the initial stages of reading, with a focus on both letter recognition accuracy and speed. To address this aim, the study is guided by the following research questions:

1- Does the use of embedded picture mnemonics have a significant effect on students’ letter recognition accuracy and associated sounds during the initial stages of reading?
2- Does the use of embedded picture mnemonics have a significant effect on students’ letter recognition speed during early reading instruction?

## 2 Method

### 2.1 Research design

The study aimed to investigate the impact of embedded picture mnemonics, developed in a previous study co-authored by the researcher (Kalender & Guleç, 2021), on letter recognition and recall in first-grade reading. To accomplish this goal, three different first-grade classes were selected. One class served as the experimental group, receiving the intervention program, while the other two classes followed the standard curriculum without intervention. The study employed a quasi-experimental design to demonstrate the effectiveness of the mnemonics.

Among the students enrolled in first grade at a primary school during 2021–2022 academic year, teachers are assigned to the classes where the students have been distributed randomly by the school administration. According to Gliner et al. (2009), research studies lacking control over group assignment which requires utilizing already distributed groups are categorized as quasi-experimental research. In this study, the absence of control over group assignment and the requirement to work with pre-organized groups necessitated the adoption of a quasi-experimental design.

While the initial random assignment of students to classrooms by the school administration provided an objective foundation, this process occurred independently of the present research and without researcher control. As a result, the study does not meet the criteria for a true experimental design. A true experimental approach was also unfeasible due to ethical and administrative constraints, particularly the need to preserve existing classroom structures and avoid disrupting instructional continuity. Consequently, the study employs naturally occurring groups within a quasi-experimental framework, which is a widely accepted methodological approach in educational field research where researcher-led randomization is not feasible.

### 2.2 Participants

This study utilized a quasi-experimental design consisting of one experimental and two control groups to investigate the effectiveness of picture mnemonics in teaching letter recognition and recall. The participant pool consisted of 95 first-grade students enrolled in a public primary school during the 2021–2022 academic year. After the initial screening process, 85 students (Experimental group: *n* = 30; Control Group 1: *n* = 25; Control Group 2: *n* = 30) were included in the final analysis.

Each group represented a different classroom taught by a separate primary school teacher, which reflects the typical structure in the Turkish primary education system—each classroom has a dedicated teacher responsible for all instructional activities throughout the academic year. To address potential teacher-related variability, two control groups were included. This design enabled comparisons between the experimental group and two distinct instructional environments, which was intended to strengthen internal validity by allowing comparisons across different instructional contexts.

All students came from similar socioeconomic and sociocultural backgrounds, were between 72 and 78 months old, and had attended preschool. Prior to the intervention, students’ initial letter knowledge was examined through a screening process involving a recognition task with the 29 letters of the Turkish alphabet. Students were asked to identify and name each letter, and those demonstrating prior letter knowledge (2 in the experimental group, 5 in Control Group 1, and 3 in Control Group 2) were excluded from the study. A formal pre-test was not administered because the study focused on the very initial stage of reading instruction, where students were expected to have no established letter knowledge. Instead, baseline equivalence was ensured through this screening procedure and through the administration of a phonological awareness assessment—widely recognized as a prerequisite for reading readiness—which confirmed comparable levels across all groups. This procedure ensured that all participating students entered the study with comparable initial literacy readiness.

**Figure 4:**
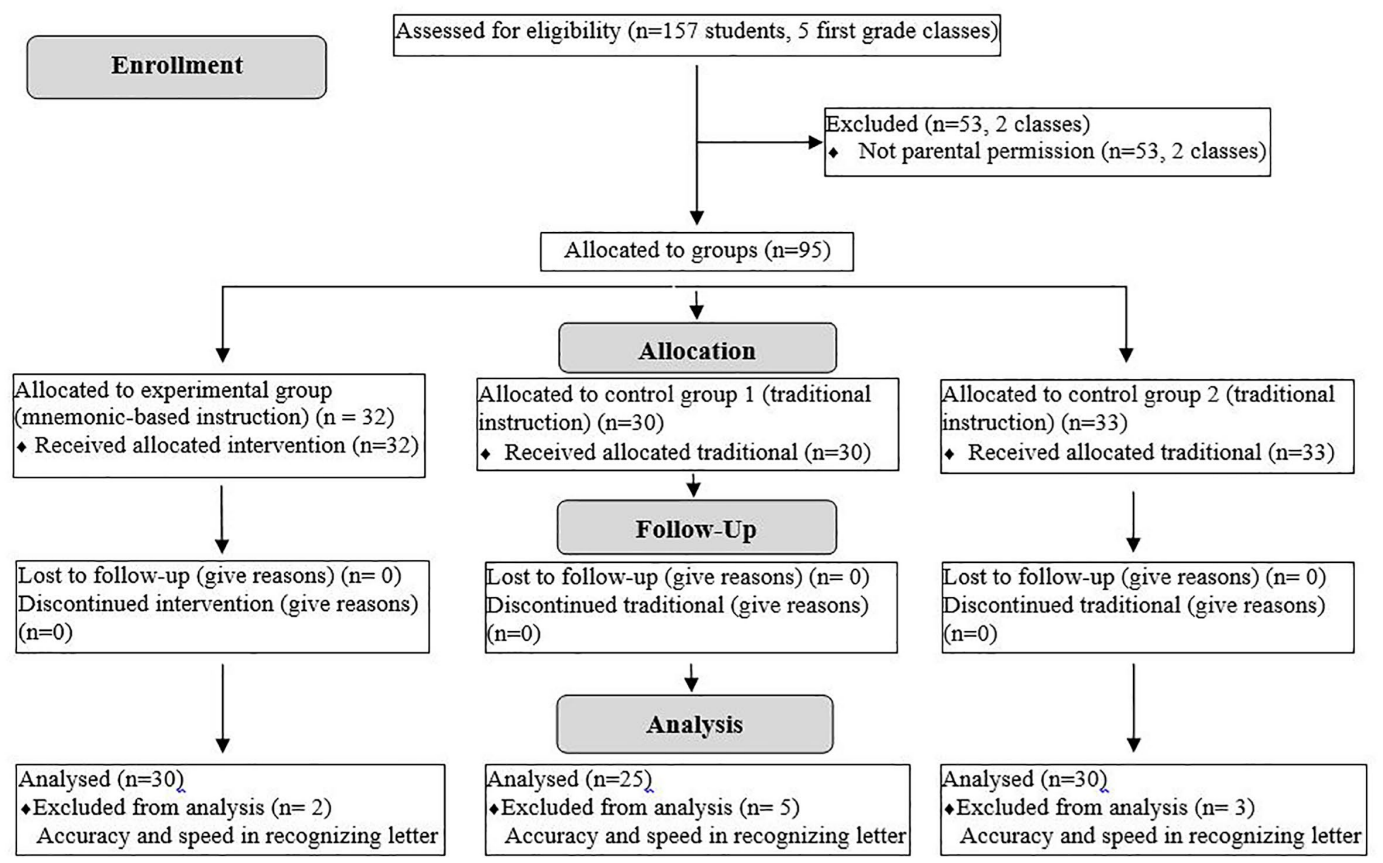
CONSORT flow diagram illustrating participant allocation in a quasi-experimental design.

Figure 4 presents a CONSORT-style flow diagram summarizing the selection and retention process of participants throughout the study.

### 2.3 Intervention procedure

The intervention spanned an 18-week period and was structured in alignment with the natural progression of literacy instruction in first-grade classrooms. During the initial 14 weeks, two new letters were introduced each week using embedded picture mnemonics, resulting in a total of 28 letters. In the 15th week, the final remaining letter was taught, and the remaining time was allocated for reinforcement activities. Weeks 16 to 18 were dedicated to general review and natural reading practices, during which the use of mnemonics was gradually phased out.

For each letter, approximately two instructional days were allocated. On the first day, the picture mnemonic was introduced, emphasizing the visual, phonetic, and associative link between the letter and a familiar object or image. On the second day, students engaged in practice activities designed to reinforce letter recognition through games, visual matching tasks, and interactive exercises. As students progressed and began forming syllables and words, the instructional emphasis shifted from individual letter recognition to more complex literacy skills. At this stage, the mnemonics were no longer the primary instructional tool; instead, they served as transitional scaffolds until students demonstrated confident recognition of standard letter forms.

The intervention was implemented by the classroom teacher, who had previously participated in the development process of the mnemonic materials reported in an earlier study (Kalender & Guleç, 2021). Prior to the intervention, the teacher received structured training on both the rationale behind the mnemonic approach and the practical aspects of its implementation. This training covered the theoretical foundations of picture mnemonics, the specific letter-mnemonic associations used in the study, and the day-to-day instructional procedures. As the teacher was directly involved in the creation of the mnemonics before the study, she was already familiar with their intended use and pedagogical purpose. Her active role throughout both the design and implementation phases ensured fidelity to the intervention protocol and contributed to the ecological validity of the study.

Importantly, the picture mnemonics were not used indefinitely. As students gained confidence and internalized the phoneme-grapheme correspondences, the visual aids were gradually withdrawn. This transition was not abrupt; initially, the mnemonic was shown, and once the student successfully recognized and articulated the letter, the standard alphabetic representation was introduced. In later weeks, as students became more familiar with the mnemonic strategy and the majority of letters, the reliance on visuals decreased. This shift allowed for increased focus on blending sounds, forming syllables, and constructing words, all of which required deeper phonological processing and literacy engagement.

The instructional sessions were embedded within the regular literacy block of the school day, typically spanning 5 days per week, with each session lasting 40 min (10 lesson hours per week). This integration ensured that the intervention was both contextually relevant and consistent with the classroom routine, thereby maximizing its feasibility and applicability in real-world educational settings. The process mentioned is presented in Table 1.

**Table 1:** The intervention process.

Week(s)	Focus	Description
Weeks 1–14	Teaching 2 letters per week using mnemonics	Total of 28 letters covered using visual, auditory, and associative cues.
Week 15	Final letter + review	29th letter introduced; initial reinforcement activities conducted.
Weeks 16–18	Consolidation and natural reading	Gradual removal of mnemonics; focus on fluent reading and transfer skills.

In accordance with the curriculum, all first-grade classrooms received the same weekly literacy instruction time (10 lesson hours per week). Thus, instructional exposure and time-on-task were structurally equivalent across groups.

### 2.4 Data collection

In this study, researcher implemented embedded picture mnemonics to support letter recognition, based on principles outlined by Ehri (2013). These mnemonics associate the phoneme of the target letter (e.g., /s/) with a visual cue resembling the shape of the letter (e.g., snake for “S”). The specific mnemonics used were developed by Kalender and Guleç (2021), and were embedded directly into the form of each letter to strengthen sound-shape associations (see Figure 2).

The intervention lasted 18 weeks. During this period, the experimental group received mnemonic-based instruction embedded into daily reading lessons. In contrast, control groups followed traditional methods, introducing letters with associated words and generic visuals (e.g., “elma” with a picture of an apple). Once students in the experimental group showed recognition and understanding of letters through mnemonic supports, the instruction transitioned to the standard letter forms without visuals.

#### 2.4.1 Measurement tools and assessment timeline

**Figure 5:**
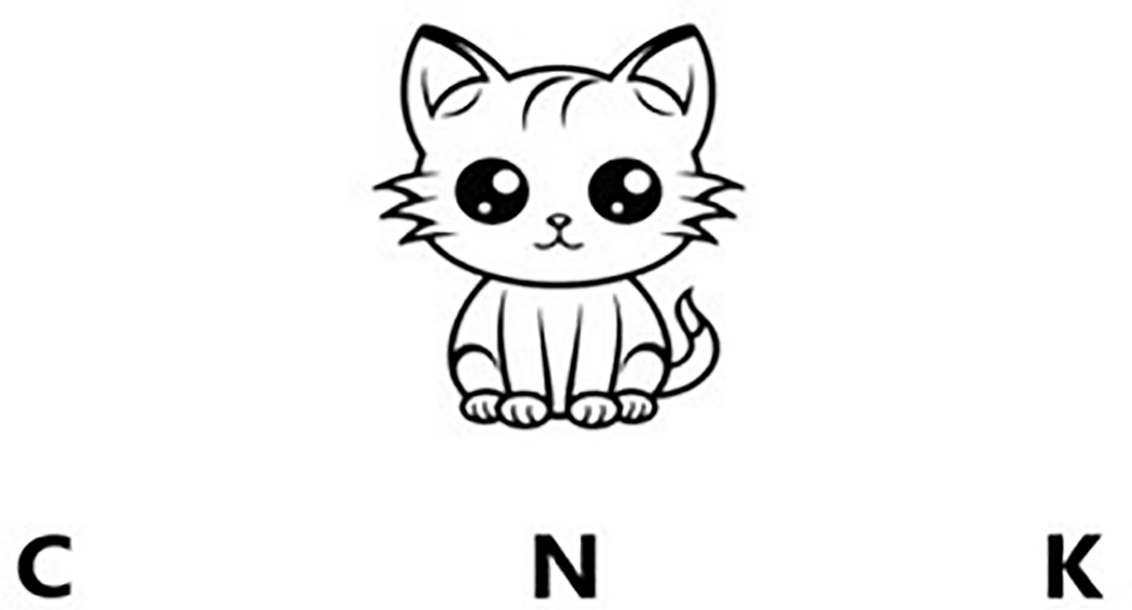
An example of the implemented test.

As McKenzie et al. (2018) suggested in their work to assess letter recognition accuracy and speed, a tablet-based multiple-choice test was developed. Each test item displayed an object image and prompted students to choose the correct initial letter from three letter options. The development and evaluation of the “A to Z Safari” iPad application demonstrated that early years literacy apps can enhance letter-sound learning through engaging gameplay and teacher support, although improvements to functionality are needed for broader classroom implementation. For instance, if the picture showed a “cat” (kedi), selecting the letter “k” was the correct answer. The application automatically recorded both the number of letter recognition accuracy and speed for each item (see Figure 5).

The assessment was designed as a task-based performance measure rather than as a psychometric scale. Accordingly, each item targeted a specific letter–sound correspondence, and performance was evaluated through observable outcomes, namely letter recognition accuracy and speed. The test consisted of 20 questions and was administered twice during the instructional period:

- Week 10: To assess students’ midterm progress after substantial exposure to the intervention.
- Week 18: To evaluate final outcomes following the completion of the initial reading instruction phase.

These assessments were not part of the intervention but served as measurement points to evaluate both short- and long-term impacts. Accordingly, the assessments followed a curriculum-based measurement approach, whereby each measurement point reflected the instructional content students had been exposed to at that stage, rather than aiming for psychometric equivalence across time points.

#### 2.4.2 Development and validation of the tool

The test design was reviewed by four field experts in early literacy, educational psychology, and cognitive development to ensure construct validity and age appropriateness. Following expert feedback, the test underwent two pilot trials to finalize item number, timing, and digital interface suitability for young children.

Prior to each administration, students were given brief instructions and allowed to practice the interface. The testing session was conducted individually using tablets, and the software automatically recorded each student’s letter recognition accuracy and speed, operationalized as indicators of recognition and processing fluency. Given the performance-based nature of the task, reliability was addressed through standardized administration procedures rather than internal consistency estimates. All students completed the task individually under identical conditions using the same digital interface, instructions, and automated scoring system. This procedure minimized administrator- and scorer-related variability and ensured consistency in data collection across measurement points. This approach is commonly adopted in early literacy research employing performance-based measures of letter–sound knowledge (McKenzie et al., 2018; Neumann, 2016; Neumann and Neumann, 2019).

### 2.5 Data analysis

To examine the effects of the mnemonic-based intervention on students’ letter recognition accuracy and speed, a 2 (Time: Week 10, Week 18) x 3 (Group: Experimental, Control 1, Control 2) doubly multivariate repeated measures analysis of variance (RM-MANOVA) was conducted (O'Brien and Kaiser, 1985). This statistical method was selected to evaluate interaction effects between time (post-I – post-II measurements) and group (experimental vs. control groups) on the two dependent variables simultaneously. Prior to conducting the RM-MANOVA, the assumptions of normality, homogeneity of variance–covariance matrices, and sphericity were tested to ensure the appropriateness of the analysis. Where necessary, adjustments (e.g., Greenhouse–Geisser correction) were applied. When significant multivariate effects were detected, follow-up univariate tests provided within the RM-MANOVA framework and Bonferroni-adjusted pairwise comparisons were examined to identify the source of these effects.

To address statistical power considerations, a sensitivity power analysis was conducted using G*Power 3.1. With *α* = 0.05, power (1 − *β*) = 0.80, three groups, and a total sample size of *N* = 85, the minimum detectable effect size was *f* = 0.34. The observed effect sizes for both dependent variables were above this value.

In addition to inferential statistics, descriptive analyses (means and standard deviations) for each group at both measurement points were reported to provide a comprehensive understanding of performance trends in letter recognition accuracy and speed.

### 2.6 Ethical considerations

This study received ethical approval from the Social and Human Sciences Ethics Committee of Gaziantep University. The research was conducted in accordance with the principles of the Declaration of Helsinki. The research was deemed ethically appropriate in the committee’s meeting dated August 3, 2022 (Meeting No: 09, Decision No: 16; Document No: E-73628654-604.01.01-219457). Prior to data collection, informed consent was obtained from students’ parents, and both the students and their teachers were provided with detailed information about the study. Participation was voluntary. All personal data were kept confidential and were not shared with any third party outside the scope of the research.

## 3 Results

### 3.1 Results related to descriptive statistics

Since the students had no prior knowledge of letters at the beginning of the intervention, a pre-test was not feasible. Therefore, the first data collection point was determined as the 10th week (Post-test I), by which time all groups had been introduced to a comparable number of letters. The second measurement was conducted at the end of the 18th week (Post-test II), after the full introduction of the Turkish alphabet. These time points allowed for examining performance at two instructionally meaningful stages of letter instruction.

Throughout the intervention program, mnemonics integrated into technological tools was administered to the experimental group for the recognition and recall of the letters. This process lasted for 18 weeks, and in the 10th week of the implementation, data were collected from both the experimental and control groups on letter recognition accuracy and speed. At the first measurement point (Week 10), the assessment was administered using only those letter items that had been fully introduced and practiced in all three groups, in order to ensure equivalent content exposure and avoid bias arising from minor differences in instructional pacing (approximately one letter). At the second measurement point (Week 18), data collection was conducted after all groups had completed instruction on the full set of 29 letters. Although minor timing differences of approximately 1 week existed across groups in completing letter instruction, the assessment was administered on the same week and day for all groups to minimize potential timing-related confounds. At the end of the 18th week, when all groups had completed the full alphabet, a set of 20 letters was selected for the second-cycle measurement (out of the 29 letters in the Turkish alphabet), primarily drawing on letters introduced after the 10th week, in alignment with the instructional content covered at each measurement point. Descriptive statistics for the results of these two measurements are presented in Table 2.

**Table 2:** Descriptive statistics regarding letter recognition accuracy and speed scores of the experimental and control groups.

Group	N	Time I (10th week post-test)				Time II (18th week post-test)			
		Accuracy		Speed		Accuracy		Speed	
		M	SD	M	SD	M	SD	M	SD
Experimental	30	18.43	2.19	168.13	69.64	19.53	1.01	143.77	59.55
Control 1	25	17.52	2.40	275.80	105.32	17.64	2.63	244.92	74.43
Control 2	30	16.20	4.09	275.63	98.16	15.70	4.78	256.40	99.14

**Figure 6:**
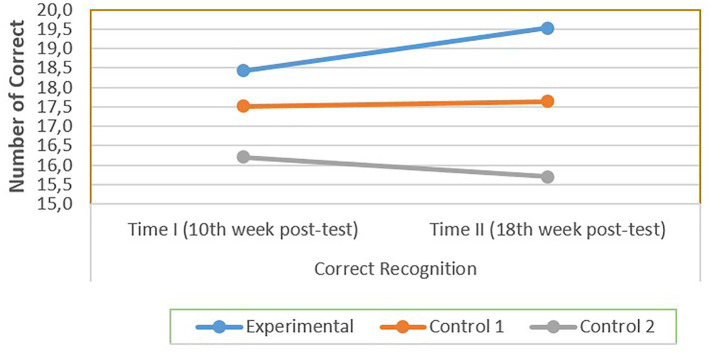
A comparative performance of experimental and control groups in letter recognition. These scores were collected after 10 and 18 weeks of instruction. No pre-test was conducted due to student’s initial lack of letter knowledge.

**Figure 7:**
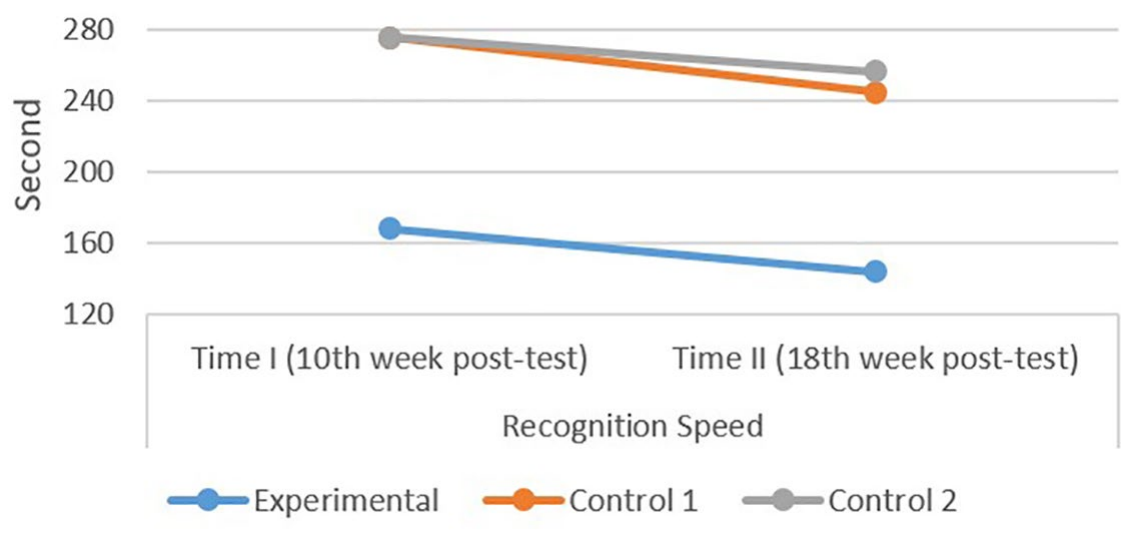
A comparative performance of experimental and control groups in recognition speed. These scores were collected after 10 and 18 weeks of instruction. No pre-test was conducted due to student’s initial lack of letter knowledge.

As displayed in Table 2, the experimental group outperformed the control groups descriptively in terms of both letter recognition accuracy and speed in both measurements. In terms of showing this change, Figures 6, 7 show the difference between groups and between the measurements.

As represented in Figure 6, the experimental group consistently showed a higher letter recognition accuracy than both control groups in both measurements. The experimental group demonstrated higher accuracy levels at both measurement points.

As shown in Figure 7, all groups demonstrated faster letter recognition speed over time; however, the experimental group demonstrated consistently faster recognition speed across both measurement points.

Notably, despite all groups starting with no prior letter knowledge, the 10-week intervention was sufficient for the experimental group to outperform both control groups in the first measurement. Group differences observed at the first measurement point were descriptively similar at the second measurement. These findings should be interpreted within the specific context of one public primary school in Türkiye, involving first-grade students learning a relatively transparent orthography.

### 3.2 Inferential analysis and assumption checks

To further investigate the research questions, a 2 (Time) × 3 (Group) doubly multivariate RM-MANOVA was employed. This statistical approach enabled a simultaneous evaluation of both in letter recognition accuracy and speed, while effectively controlling for the intercorrelation between these two variables and mitigating the risk of Type I error inflation.

Prior to the main analysis, the distributional characteristics of the data were examined. Univariate normality was assessed separately for each dependent variable (letter recognition accuracy and speed) at both measurement points using Shapiro–Wilk tests and distributional indices. The results indicated significant departures from normality across groups (*p* < 0.05), characterized by pronounced negative skewness and kurtosis, particularly at the final measurement. Although some measures showed a typical skewness due to the expected ceiling effect—where students reached high mastery levels by the final assessment—these deviations were considered theoretically meaningful and manageable within the RM-MANOVA framework, particularly given the use of Pillai’s Trace as a robust test statistic. For the RM-MANOVA results, Box’s M value was found to be significant (*p* < 0.05); therefore, Pillai’s Trace was used to interpret multivariate effects (Field, 2013). No cases were excluded from the analyses following outlier screening. Taken together, the analytical approach was considered appropriate for the structure and characteristics of the data, given the robustness of Pillai’s Trace to violations of multivariate normality and variance–covariance homogeneity.

The RM-MANOVA revealed a significant main effect of Group [Pillai’s V = 0.438, *F*(4, 164) = 11.51, *p* < 0.001, ηp^2^ = 0.219], confirming that the instructional methods led to distinct overall performance profiles. The multivariate main effect of Time was marginally significant [Pillai’s V = 0.064, *F*(2, 81) = 2.78, *p* = 0.068, ηp^2^ = 0.064], while the Time * Group interaction did not reach statistical significance (*p* = 0.628). The comprehensive results of the doubly multivariate RM-MANOVA, including the multivariate tests and their follow-up univariate analyses, are summarized in Table 3.

**Table 3:** Doubly multivariate RM-MANOVA results for letter recognition accuracy and speed.

Source of variance	Variable	Sum of squares	df	Mean squares	*F*_(1, 82)_	*p*	η_p_^2^	Difference (Bonferroni)
Time	Accuracy	2.43	1	2.43	0.31	0.582	0.004	
	Speed	26002.83	1	26002.83	5.45	0.022*	0.062	T2 > T1
Time*Group	Accuracy	19.49	2	9.74	1.23	0.299	0.029	
	Speed	925.10	2	462.55	0.10	0.908	0.002	
Group	Accuracy	276.49	2	138.24	11.68	0.000*	0.222	Exp. > C2
	Speed	449489.14	2	224744.57	22.59	0.000*	0.355	Exp. > C1, C2
Error	Accuracy	970.51	82	11.84				
	Speed	815609.35	82	9946.46				

Bold values indicate statistically significant effects (*p* < 0.05).

#### 3.2.1 Results related to RQ1: effect of mnemonics on letter recognition accuracy

The first research question examined whether the use of embedded picture mnemonics significantly affected students’ letter recognition accuracy. Follow-up univariate tests (see Table 3) showed a significant main effect of Group on accuracy [*F*(2, 82) = 11.68, *p* < 0.001, ηp^2^ = 0.222]. Bonferroni post-hoc comparisons indicated that the experimental group significantly outperformed Control Group 2 (*p* < 0.001). However, no significant difference was observed between the experimental group and Control Group 1 (*p* = 0.108). This result suggests that while the mnemonic intervention facilitated high accuracy, the widespread ceiling effect—where students across all groups achieved mastery by the 18th week—narrowed the performance gap between the experimental group and the traditional instruction group (C1) on this particular measure.

#### 3.2.2 Results related to RQ2: effect of Mnemonics on letter recognition speed

The second research question focused on whether mnemonic instruction influenced letter recognition speed. Univariate test results revealed a highly significant and substantial main effect of Group on recognition speed [*F*(2, 82) = 22.59, *p* < 0.001, ηp^2^ = 0.355]. Pairwise comparisons confirmed that the experimental group was significantly faster in recognizing letters than both Control Group 1 (*p* < 0.001) and Control Group 2 (*p* < 0.001). Furthermore, a significant main effect of Time was found for recognition speed [*F*(1, 82) = 5.45, *p* = 0.022, ηp^2^ = 0.062], indicating that students across all groups significantly improved their retrieval fluency between the midterm and final assessments. The large effect size for the group factor (ηp^2^ = 0.355) underscores the relative effectiveness of the intervention. This result highlights that the visual-phonetic links provided by the mnemonics were instrumental in fostering automaticity in letter-sound retrieval—a critical step toward reading fluency that clearly differentiated the experimental group from traditional classroom environments.

## 4 Discussion and conclusion

The findings of the present study demonstrate the effects of embedded picture mnemonic strategies on first-grade students’ letter recognition skills across two core dimensions: accuracy and speed. The results were interpreted within the frameworks of CLT (Sweller, 1988; Sweller et al., 2011) and DCT (Paivio, 1986, 1991), and discussed in relation to prior research as well as the orthographic characteristics of the Turkish language.

One of the key findings of the study is that the experimental group demonstrated a statistically significant advantage over the control groups in letter recognition accuracy at the 10th week of instruction. Although this difference narrowed statistically by the 18th week, the experimental group maintained its relative advantage over the control groups. This pattern is consistent with previous research indicating that embedded picture mnemonics accelerate the establishment of letter–sound relationships, particularly during the early phases of learning (Ehri, 2005; Fulk et al., 1997; Agramonte and Belfiore, 2002). The reduction in group differences observed at the 18th week can be explained by the phenomenon commonly referred to as the ceiling effect. Due to the phonological transparency of Turkish and the high regularity of letter–sound correspondences, children tend to reach high levels of accuracy relatively quickly as instruction progresses (Öney and Durgunoğlu, 1997).

According to the orthographic depth hypothesis, accuracy-based measures in transparent orthographies such as Turkish tend to reach saturation at an early stage, making it more difficult to distinguish cognitive differences between groups using accuracy alone (Borleffs et al., 2019). At this point, as emphasized by Ehri (2005) and Ehri et al. (1984), the primary goal of letter–sound instruction is not merely correct recognition, but the unitization of letter–sound knowledge and its effortless retrieval. Shmidman and Ehri (2010) argue that embedded picture mnemonics strengthen this unitization process by making letter–sound connections more durable and cognitively accessible. From this perspective, the similarity observed in accuracy scores at the 18th week does not indicate that learning quality has converged across groups; rather, it suggests that accuracy measures become limited in their ability to capture differences in levels of automaticity. Indeed, as noted by Kargın et al. (2024), in highly transparent languages such as Turkish, reading success is primarily differentiated by speed and fluency rather than accuracy. Accordingly, the recognition speed findings of the present study provide a more sensitive and theoretically meaningful indicator of instructional impact.

Another major finding of the study is the large effect of embedded picture mnemonics on letter recognition speed (ηp^2^ = 0.355). The ability to convert a visual letter into its corresponding sound immediately is a foundational component of reading fluency. In their study conducted in Hebrew, a language with a more complex orthography, Shmidman and Ehri (2010) demonstrated that embedded picture mnemonics enhanced the retention of letter–sound connections. The present study provides context-specific evidence within the Turkish orthographic system, suggesting that the strategy may support retention and retrieval fluency under conditions of high phonological transparency. Because letters in Turkish typically correspond to a single sound, embedded picture mnemonics appear to facilitate the integration of a letter’s visual form and its phonological representation into a single cognitive unit. As a result, children are less likely to rely on an additional translation process during letter–sound mapping. As emphasized in Mayer’s (2001) Cognitive Theory of Multimedia Learning, the simultaneous and integrated presentation of verbal and visual information enhances both learning efficiency and retention. When combined with the phonetic clarity of Turkish, this multimodal integration provides a compelling explanation for the substantial speed advantage observed in the experimental group.

The findings also yield important implications when interpreted through the integrated lenses of CLT and DCT. According to Sweller (2011), literacy skills such as reading and writing constitute biologically secondary knowledge and therefore require carefully designed instructional support. In traditional instructional approaches, letter–sound relationships are often presented in abstract or arbitrary ways, which can impose unnecessary extraneous cognitive load on working memory (Houston, 2025). In contrast, the embedded picture mnemonics used in this study may operate in a manner consistent with a reduction in extraneous cognitive load by transforming the abstract letter form into an inseparable component of a meaningful visual representation. Presenting the letter, sound, and image as a single integrated structure may reduce the need for learners to mentally combine disparate elements, thereby enhancing instructional efficiency (Sweller, 2011; Paas and van Merrienboer, 1994). Consistent with McNamara’s (2012) findings, embedded mnemonic cards enable faster and more durable acquisition of letter–sound knowledge compared to approaches in which letters and images are presented separately. From the perspective of Paivio’s (1991) dual-channel processing model, the concurrent and complementary activation of verbal and visual systems provides a robust explanation for the automaticity observed in the experimental group. Drawing on Paas et al.’s (2003a) assertion that high performance achieved with lower cognitive effort reflects instructional effectiveness, the mnemonic-based strategy employed in this study can be interpreted as theoretically aligned with CLT principles. However, as no direct cognitive load measures (e.g., mental-effort ratings or physiological indicators) were collected, these interpretations should be understood as theoretically informed rather than empirically verified mechanisms.

In addition, certain instructional practices commonly adopted during early reading instruction may inadvertently hinder learning. Prior research indicates that instructional approaches emphasizing letter names rather than letter sounds can complicate the development of accurate letter–sound mappings, particularly when deductive teaching dominates early instruction (Rony, 2023; Treiman et al., 2008). Although letter-name knowledge may support letter–sound learning for some children, its benefits are not uniform, and an overreliance on name-focused instruction has been shown to delay phonemic sensitivity and automatic sound mapping (McGuinness, 2004; Piasta et al., 2010). The visually grounded approach implemented in this study resembles the Picture–Word Inductive Model described by Jiang and Perkins (2013), facilitating the placement of abstract symbols into meaningful mental schemas. As noted by Shaeffer (2011), visually supported mnemonic strategies promote not rote memorization but the meaningful and durable encoding of letter–sound knowledge in long-term memory.

Several potential validity threats also warrant consideration. Teacher effects and the use of digital tools such as tablets—which may influence student engagement—represent important factors in interpreting the findings. The possibility of teacher effects was considered through the inclusion of two control groups taught by different teachers, a cautious examination of this influence. Consistent with the logic underlying unrelated design approaches (Randler and Bogner, 2008), the inclusion of multiple teachers allowed for a more cautious consideration of whether the observed effects were tied to the instructional strategy rather than to a single teacher’s instructional style. Nevertheless, it is not possible to claim that teacher effects were entirely eliminated. Comparing three teachers working with demographically similar classrooms helped contextualize potential teacher-related variance and highlight the effectiveness of the embedded picture mnemonic strategy. As also observed by Vaughn et al. (2016), well-structured interventions can yield comparable learning gains even when implemented by different practitioners.

The potential motivational effects of tablet-based assessment should also be acknowledged. Brüggemann et al. (2023) reported that digital environments may initially enhance student motivation, although this effect tends to stabilize over time. This pattern may be applicable to the present study. Importantly, all groups were assessed under identical conditions using the same digital tools, which likely prevented differential motivational effects from confounding group comparisons and strengthened the internal validity of the findings. Moreover, Salmerón et al. (2021) demonstrated that tablet use does not negatively affect task-focused attention at the primary school level. Therefore, the pronounced speed advantage observed in the experimental group is better explained by the cognitive qualities of the embedded visual mnemonic strategy rather than by novelty or technology-related interest alone.

In conclusion, this study provides empirical evidence that embedded picture mnemonics can function as an effective instructional support for enhancing letter–sound awareness within a Turkish first-grade context. The findings indicate that this approach may support both accurate letter recognition and retrieval speed. However, given that the study was conducted in a single school, within one language system, and with a specific age group, the results should be interpreted cautiously and not generalized beyond comparable instructional and linguistic contexts. Future research across different orthographies, age groups, and educational settings is needed to determine the broader applicability of the approach.

### 4.1 Limitations and recommendations

#### 4.1.1 Methodological and scope-related limitations

Despite its contributions, this study has several limitations that should be considered when interpreting the findings.

- The quasi-experimental design based on naturally occurring classroom groups may have introduced selection effects that could affect internal validity. Although baseline equivalence was supported through letter-knowledge screening and phonological awareness assessments, unmeasured classroom-level factors may still have played a role.
- A ceiling effect in letter recognition accuracy at the final assessment point may have limited the sensitivity of this measure to detect group differences over time.
- Gains in recognition speed may partially reflect maturation and increasing familiarity with the task, in addition to the instructional intervention.
- In addition to ceiling effects and maturation, an important methodological limitation concerns the use of different letter sets at Post-test I (Week 10) and Post-test II (Week 18). Because the assessments were aligned with the instructional content covered at each stage, the two measurement points were not designed to be psychometrically equivalent. While this curriculum-based measurement approach ensured that students were not assessed on letters they had not yet been formally introduced to, it also limits the strength of strict longitudinal comparisons across time. Therefore, changes observed between Post-test I and Post-test II should be interpreted with caution, and the findings are best understood as reflecting group differences at each instructional stage rather than precise growth trajectories over identical item sets.
- The study was conducted in three intact classrooms. Due to the small number of clusters, classroom-level effects could not be modeled using multilevel analysis. Therefore, potential teacher or classroom influences cannot be fully disentangled from the intervention effect and should be interpreted with caution.
- Although multiple teachers were involved, the teacher of the experimental group co-developed the instructional materials (Kalender & Guleç, 2021) and received targeted training prior to implementation. No independent fidelity observations, structured adherence monitoring, or blinded outcome assessments were conducted. Although instructional time was equivalent across classrooms in accordance with the curriculum weekly literacy schedule, potential allegiance and expectancy effects cannot be ruled out as alternative explanations for part of the observed gains. Future research should incorporate independent fidelity monitoring and blinded assessment procedures to more rigorously isolate the effects of the instructional strategy itself.
- Finally, the performance-based assessment emphasized standardization rather than internal consistency estimates, which may limit direct comparisons with scale-based measures.

These considerations may inform future studies using randomized designs and more sensitive outcome measures.

#### 4.1.2 Implications for educational practice and future research

Despite these limitations, the results provide meaningful implications for early literacy instruction. The strong group differences observed in recognition speed suggest that embedded picture mnemonics may be particularly effective in supporting the development of automatic letter–sound retrieval. For classroom practice, this highlights the potential value of integrating visually meaningful mnemonic supports during the early phases of reading instruction, especially for students who require additional support in fluency development. Teacher education and in-service training programs may benefit from explicit guidance on how mnemonic-based strategies can be systematically incorporated into early literacy curricula.

Future research could build on the present results in several ways. Studies using randomized designs and larger samples would strengthen causal interpretations. Further investigations might examine the effectiveness of individual mnemonics for specific letters, allowing for more fine-grained instructional design. Longitudinal research could explore whether early gains in letter recognition speed are sustained and transferred to later reading outcomes, such as word decoding and reading comprehension. Such extensions would contribute to a more comprehensive understanding of the role of mnemonics in early reading development.

## Data Availability

The datasets presented in this study can be found in online repositories. The names of the repository/repositories and accession number(s) can be found at: https://zenodo.org/records/16976421?token=eyJhbGciOiJIUzUxMiJ9.eyJpZCI6IjM1Y2M4NzkxLTY0ZGMtNGYyYy04YzI5LWViNjdjN2U0ZDVkOSIsImRhdGEiOnt9LCJyYW5kb20iOiIxZTEzYzIyMDhkZTc2OTEwZjZiZmZkZmVhOGQxM2QxOSJ9.qI0AXCIgOg21wGrSp0yQtSYDUEJMWORGP3BRI0jbIBILb2J4dmKhIKM7sZ_dlX6-fm7hUZ8nP9MIhc-MA0zsGw.
